# Supplementary material for: ChromActivity: integrative epigenomic and functional characterization assay based annotation of regulatory activity across diverse human cell types
Source: Genome Biol. 2025 May 9;26:123. doi: 10.1186/s13059-025-03579-6 (PMC12063466; doi:10.1186/s13059-025-03579-6)
Supplement: Supplementary file 2 — Additional file 2. Review history. [file 13059_2025_3579_MOESM2_ESM.docx]

**1^st^ round**

**Reviewer 1**

Dincer and Ernst developed a computational model called ChromActivity to predict the regulatory activity of a genomic element by integrating histone modification ChIPseq data and readouts obtained from reporter or perturbation essays. They rigorously evaluated this method and showed that the performance is good in general. Interestingly, the authors also noted a number of factors affecting the performance, such as the specific functional assay being incorporated and the cell-type in which the prediction model is built from. Using this model, they created a genomewide regulatory activity prediction map, called ChromScore, in 127 cell types that are previously profiled by the Epigenome Roadmap and ENCODE consortium. In addition, they also adapted the ChromHMM model Dr. Ernst previously developed to analyze the ChromActivity output, and referred to the outcome as ChromScoreHMM. Overall, this is a nicely carried out study providing a valuable resource to the community. My specific comments are below:
Major Comments:
1. The biological interpretation of the ChromScoreHMM model output is not clear. Aside from enrichment analysis of certain well-characterized genomic elements, which can be done by using the traditional ChromHMM and similar tools already, more evidence is needed to convince people this is useful. In fact, Figure 4B suggests that the benefit of ChromScore analysis is limited. Although it performs better than several existing models, it is comparable to the simple approach using DNase-I hypersensitivity signal for prediction.
2. It is perhaps not surprising that ChromScore prediction is only moderately correlated with gene expression, since it does not use any DNA sequence information. However, I was also wondering if focusing too much on TSS surrounding regions is part of the reason, since distal regulatory elements are well-known to play an important role. Is it possible to incorporate distal information into the ChromScore framework as well?
3. It is not clear if the ChromActivity and ChromScoreHMM models are publicly available with tutorials so that users can run their own analyses.
Minor Comments:
1. It is interesting that the predictions are somewhat sensitive to the source of functional assays. It would be interesting if the authors can comment on the potential reasons. On the other hand, it is somewhat concerning that predictions based on different assays can are negatively correlated in some cases. Rather than simply combining the outcomes, it might be cautious to further investigate the underlying cause to either refine the models and/or provide an estimate of uncertainty.
2. The models generated by different functional data sources are surprisingly low in some cases (e.g. between Ernst/MPRA/K562 and Kheradpour/MPRA/K562, and less so between Fulco/CRISPR/K562 and Gasperini/CRISPR/K562). Does this reflect the intrinsic sensitivity of the prediction models?

**Reviewer 2**

The authors present a new approach for the prediction of active regulatory regions by integrating several types of genome-activity assays in a supervised framework, in which histone mark data is used as features for regression. The ChromScore and subsequent ChromScoreHMM is designed as a way to integrate and interpret histone marks in regulatory regions. The authors present interesting results regarding their new approach and compare to other existing methods that score regions of relevance as well as a thorough analysis of their new predictions. Albeit complex, the paper is generally well written.

Major comments:
- The authors use imputed signal tracks for 10 histone marks and Dnase1 for the learning of the ChromActivity epxerts. In how far do the results differ if real tracks are being used? Is the prediction performance similar for imputed and real histone tracks?

-As the authors use so many tracks as input I wasn't able to understand, how the method could be used by others? Does a user always have to give the histone, DNase and CHrommHMM state tracks as input? Are all of those features equally important? It is quite a lot of input features that a user would have to prepare.

- In the context of the evaluation and comparison of ChromScore performance (Fig 4B) I would be interested to understand better how the difference between CRISPR and Plasmid based assays can be understood. In Supp Fig S15 A, it shows that ChromScore works relatively poor for CRISPR-based assays in comparison to many other established methods and even the single histone tracks. How can this be? For Plasmid based assays (SUpp Fig S15 B, it looks quite different).

-Similarly Fig 4B shows that the individual DNase1 track alone works almost as good as the complete ChromScore approach. The authors write that the correlation with DNAse1 signal is only moderate, which may argue for some type of complementarity. It would be great if the authors could explore this in more detail. For example using true positive cases, is there something specific about the regions that are found with DNAse1 or vice versa?

- In the methods section (page 31, lines 523-525) the authors mention that they excluded repressive sequences from the training. I would like to understand the rational for this. Did it show improvement for the learning?


Minor comments:
- I find the use of the word ChromActivity as an overall name a bit confusing in the wordings sometimes. With ChromActivity the authors sometimes describe the supervised learning part of individual assays, and sometimes more generally the framework that can do many things. Maybe the authors could check the text carefully to avoid ambigous mentions (such as page 10, line 139: In addition, ChromActivity averages ...).

- Figure 2 B, denotes the names of ChromHMM states on the x-axis, but these names are not explained anywhere. Please add a description to the paper (possibly in the legend of Fig2) so that the reader can follow. Supp Fig. S2 explains the color code but not the meaning of the abbreviations. And personally I don't find it helpful to have the color code legend in the supplement.


- I was not able to understand how the Genome % (Annotations) row is defined in Figure 3B (top row above the heatmap). Please extend the Figure caption for it as the current description "percentage of the genome occupied by the annotation" is ambiguous. Currently the numbers do not add up to 100%.

- I prefer the visualisatoin in Supp Fig S1 for teh ChromActivity framework over what is currently in Fig 1A, as it contains more details and also helps to understand the difference between ChromScore and ChromscoreHMM. I suggest that the authors integrate it into the main paper.

**Authors’ response**

**Reviewer 1**

Dincer and Ernst developed a computational model called ChromActivity to predict the regulatory activity of a genomic element by integrating histone modification ChIPseq data and readouts obtained from reporter or perturbation essays. They rigorously evaluated this method and showed that the performance is good in general. Interestingly, the authors also noted a number of factors affecting the performance, such as the specific functional assay being incorporated and the cell-type in which the prediction model is built from. Using this model, they created a genomewide regulatory activity prediction map, called ChromScore, in 127 cell types that are previously profiled by the Epigenome Roadmap and ENCODE consortium. In addition, they also adapted the ChromHMM model Dr. Ernst previously developed to analyze the ChromActivity output, and referred to the outcome as ChromScoreHMM. Overall, this is a nicely carried out study providing a valuable resource to the community. My specific comments are below:

**Response:** We thank the reviewer for the summary and the positive and constructive comments. We provide a point by point response to the individual comments below.

**Major comments**

1. The biological interpretation of the ChromScoreHMM model output is not clear. Aside from enrichment analysis of certain well-characterized genomic elements, which can be done by using the traditional ChromHMM and similar tools already, more evidence is needed to convince people this is useful. In fact, Figure 4B suggests that the benefit of ChromScore analysis is limited. Although it performs better than several existing models, it is comparable to the simple approach using DNase-I hypersensitivity signal for prediction.

**Response:** We thank the reviewer for the comment. We believe ChromScoreHMM state annotations can be a useful complement to ChromHMM state annotations for certain applications, specifically those for which having a more detailed annotation of potential regulatory regions is important.

The interpretation of ChromScoreHMM states differ from ChromHMM in that ChromScoreHMM states can be explicitly interpreted to be associated with regions that are predicted to have greater activity in certain classes of testing experiments such CRISPR-dCas9 (ChromScoreHMM State 5) or STARR-seq assays (ChromScoreHMM State 10). We updated the Discussion section to address this point.

Notably, ChromScoreHMM states explicitly capture genomic regions that are more active in CRISPR-dCas9 or STARR-seq assays.

To more directly quantitatively compare the ChromScoreHMM annotations to ChromHMM annotations with orthogonal genomic data associated with regulatory activity, we used transcription factor binding peak data. The ChromHMM model was the same 25-state model that was used as input features to ChromActivity. The transcription factor binding data was from seven ENCODE cell types with extensive ChIP-seq data. For each cell type, we concatenated all the peaks and then used the center base for enrichment. We computed the cumulative fold enrichment and genome coverage when states were ordered based on enrichment for transcription factor binding for both the ChromScoreHMM and ChromScore (Supplementary Figure S13). This revealed that for six of the seven cell types, the most enriched ChromScoreHMM states had both greater genome coverage and TF enrichments than a set of most enriched ChromHMM states with the exception being H1hesc.

In the results section: “Enrichment analysis of ChromScoreHMM states” we have added:

We also evaluated the enrichment of ChromScoreHMM states for experimentally determined transcription factor binding locations matched to the cell type of the annotations, which we would expect to enrich in bases of regulatory activity, and compared it to that of a ChromHMM model. For this we focused on seven cell types with a large number of ChIP-seq experiments from ENCODE (A549, GM12878, H1hesc, HeLa-S3, Hepg2, Huvec, K562). For each cell type, we computed enrichments for the center base of peaks after combining peak calls for all experiments in the cell type considered. We analyzed the cumulative fold enrichments of peak centers relative to the cumulative genome coverage of states when the states are ordered based on their enrichment. The ChromHMM model we used was the same 25-state model that was input to ChromActivity. Despite having fewer states, for six of the seven cell lines the most enriched ChromScoreHMM annotations were collectively able to have greater enrichment for transcription factor binding peak centers while covering more of the genome than a set of most enriched ChromHMM states (Supplementary Figure S13). The one exception was H1hesc, this may be partly because of increased TF binding in these cells without being associated with regulatory activity. Consistent with that, the state associated with DNase-I hypersensitivity without any active histone modifications (19_DNase) had a 40-fold enrichment for transcription factor binding peak centers compared to between 11- and 21-fold in other cell types considered. However, some of the reduced performance in H1hesc might also be reflecting epigenome differences in this cell type relative to other cell types considered. These analyses suggest, in many cell types, advantages of ChromScoreHMM for identifying a limited set of high-confidence regulatory sites but also complementary advantages of ChromHMM in other cases.

**Cumulative transcription factor binding peak enrichments and genome coverage for ChromScoreHMM and ChromHMM annotations**. **(A)** The plot shows for A549 (E114) on the x-axis the cumulative % of the genome covered by the states and on the y-axis the cumulative fold enrichment of ChromScoreHMM states (red) and ChromHMM (gray) for the center base of transcription factor binding peaks when states are ordered based on enrichment. Points are labeled by the corresponding state. **(B-G)** The same as **A** except for (**B**) GM12878 (E116), **(C)** HeLa-S3 (E117), **(D)** H1hesc (E003), **(E)** Hepg2 (E118), **(F)** Huvec (E122), **(G)** K562 (E123).

We have also added the new subsection “Transcription factor binding enrichment analysis” to the Methods section:

**Transcription factor binding enrichment analysis**

We conducted a comparative analysis of enrichment for the center base of transcription factor binding peaks between ChromScoreHMM and ChromHMM state annotations. For this comparison, we used the annotations from the 15-state ChromScoreHMM model and the annotations from the 25-state imputation based ChromHMM model, the latter of which was input features to ChromActivity. We focused on annotations from seven reference epigenomes corresponding to seven cell lines (A549 (E114), GM12878 (E116), H1hesc (E003), HeLa-S3 (E117), Hepg2 (E118), Huvec (E122), and K562 (E123)) based on the availability extensive uniformly processed ChIP-seq data from a prior ENCODE consortium compendium [7]. Specifically we collected ChIP-seq from this directory https://hgdownload.soe.ucsc.edu/goldenPath/hg19/encodeDCC/wgEncodeAwgTfbsUniform/ which had 35 experiments for A549, 90 for GM12878, 58 for H1hesc, 64 for HeLa-S3, 77 for Hepg2, 14 for Huvec, and 150 for K562.

For each cell type considered we separately concatenated peaks for all the ChIP-seq experiments into a single file. We then used the OverlapEnrichment command of ChromHMM to compute enrichments for the concatenated peaks of each cell type both for the ChromHMM and ChromScoreHMM annotations. We used the ‘-center’ flag to have the enrichment based on the center of the base of the peak. We used the ‘-multicount’ flag to count a base multiple times in the overlap if it was the center base of multiple peaks in the concatenated file. For ChromScoreHMM enrichment we used the ‘-b 25’ to specify the annotation was at 25 bp resolution while for ChromHMM we used the default ‘-b 200’ option.

Separately for each cell type, we sorted the 15-ChromScore states based on their enrichment for the peak centers. We then plotted on the x-axis the cumulative genome coverage of the states based on this ordering. On the y-axis we plotted the cumulative fold enrichment based on the same ordering. The cumulative fold enrichment was computed by first multiplying for each state the percentage of the genome a state covers by its fold enrichment for peak centers to obtain the percentage of peak centers the state contains. The cumulative sum of those percentages was then computed. Finally, the cumulative sum of percentage of peak centers was divided by the cumulative genome coverage to obtain cumulative fold enrichments.

We also now include in the Discussion text highlighting how ChromScoreHMM can effectively be used as a high resolution relative to ChromHMM as it leverages a supervised framework:

In addition, the ChromScoreHMM annotations were defined at 25-bp resolution, which is a higher resolution than ChromHMM annotations are typically defined, which is a 200-bp resolution. A challenge with higher resolution annotations with ChromHMM is the positions with locally highest signal for histone modifications are more likely to be the positions of nucleosomes and less likely the actual regulatory bases. ChromScoreHMM by using supervised information can predict a position has greater regulatory activity even if it is not locally maximal for an input feature. This is likely reflected in the greater cumulate enrichments and genome coverage among the most enriched states for the center of transcription factor binding peaks in all but one of the cell types evaluated. The exception of H1hesc also highlighted potential complementary advantages of ChromHMM.

While it is the case that ChromScore performed similarly to DNase in the functional testing predictions, the overall correlation between them was modest (median correlation 0.26), which suggests there is complementary value. We previously explored the differences of what they are capturing in Supplementary Fig S17 (now Supplementary Fig. S18). Additionally, our new feature ablation analysis (Supplementary Figure S20A) demonstrates that removing DNase signal tracks, DNase peaks calls, and also chromatin state annotations, which were in part-based on DNase signal tracks, from the model leads to only a modest decrease in cell type generalization performance, indicating that the model's overall performance has limited dependence on DNase based features. Furthermore, the ChromScoreHMM representation is a fundamentally different representation than a score as it captures multidimensional information while a signal track can only represent unidimensional information.

We have updated the “ChromScore regulatory activity predictions” section in Results to include the feature ablation study:

Removing DNase signal tracks, DNase peaks calls, and chromatin state annotations, which were in part-based on DNase signal tracks, from the model leads to only a modest decrease in cell type generalization performance, indicating that the model's overall performance has limited dependence on DNase-based features.

2. It is perhaps not surprising that ChromScore prediction is only moderately correlated with gene expression, since it does not use any DNA sequence information. However, I was also wondering if focusing too much on TSS surrounding regions is part of the reason, since distal regulatory elements are well-known to play an important role. Is it possible to incorporate distal information into the ChromScore framework as well?

**Response:** We thank the reviewer for the question. For the gene expression analysis specifically, we elected to center our intervals around the TSS, as distance in general can be a major confounder for analyses reporting associations between distal loci and gene expression.

In general, associations between loci and genes are expected to decrease as a function of distance to the target gene, but this can be in part because the gene is less likely the actual target of the distal loci. By reporting associations as a function of distance to the TSS we are able to control for potential confounding from distance.

More broadly on the question of incorporating distal information to the ChromActivity framework, our focus has been on predicting enhancer activity independent of target genes, which is also what plasmid-based assays like MPRA and STARR-seq attempt to measure. Associating distal regulatory elements with target genes is a separate goal better addressed by other frameworks. For instance the Activity-by-contact model (Fulco *et al.*, 2019) or in a correlation based framework that we previously developed (Liu *et al.*, 2017; Ernst *et al.*, 2011). However our predictions could potentially be used as input to other frameworks to help with this task, which could be a direction to pursue in future work.

We added a sentence to the discussion about this point:

ChromActivity focuses on predicting regulatory activity independent of target genes, a potential direction for future work is to evaluate the use of its predictions as input to complementary frameworks to map distal regulatory elements to their targets.

We expanded the “Expression analyses around TSSs” subsection in Methods to better explain our focus on analyzing correlations as a function of distance to TSS.

We elected to center our intervals around the TSS, as distance to the TSS in general can be a major confounder for analyses reporting association between distal loci and gene expression.

3. It is not clear if the ChromActivity and ChromScoreHMM models are publicly available with tutorials so that users can run their own analyses.

**Response:** We thank the reviewer for the comment. We have added a tutorial for users who would like to train their own models to the Github repository. The ChromActivity model weights (for ChromScore and ChromScoreHMM) and code are also publicly available on Github at https://github.com/ernstlab/chromactivity.

We have updated the “Availability of data and materials” section to point to these resources. The ChromActivity software, model weights and links to the ChromScoreHMM and ChromScore annotations (hg19 and hg38 liftOver) for the 127 Roadmap cell types are available on https://github.com/ernstlab/ChromActivity.

**Minor comments**

1. It is interesting that the predictions are somewhat sensitive to the source of functional assays. It would be interesting if the authors can comment on the potential reasons. On the other hand, it is somewhat concerning that predictions based on different assays can are negatively correlated in some cases. Rather than simply combining the outcomes, it might be cautious to further investigate the underlying cause to either refine the models and/or provide an estimate of uncertainty.

**Response:** We thank the reviewer for this comment. We respond to this comment and the related minor comment 2 in a combined response below.

2. The models generated by different functional data sources are surprisingly low in some cases (e.g. between Ernst/MPRA/K562 and Kheradpour/MPRA/K562, and less so between

Fulco/CRISPR/K562 and Gasperini/CRISPR/K562). Does this reflect the intrinsic sensitivity of the prediction models?

**Response:** We thank the reviewer for the comment. Based on this comment and the previous comment we investigated potential reasons for the low or negative correlation in some cases including for the examples mentioned. One source contributing to lower correlations that we could isolate appears to be predictions within certain classes of genomic regions that correspond to specific chromatin states having limited representation in the training sets of specific prediction models. To test this, we analyzed pairs of expert predictions noted by the reviewer for which genomewide score correlations were surprisingly low after excluding individual or pairs of chromatin states over 30 randomly selected cell types (Supplementary Figure S4). We observed that removing particular combinations of transcription-associated chromatin states resulted in positive and higher correlations. In particular, removing the chromatin states 8_TxWk and 5_Tx5’, which had zero loci tested within the Gasperini/CRISPR/K562 dataset (Supplementary Figure S2), resulted in the largest correlation increases for pairs of datasets that included Gasperini/CRISPR/K562 (Supplementary Figure S4). We updated the “Genomewide expert predictions” subsection to include the new analysis:

We investigated potential reasons for low or negative score correlations among some pairs of experts. We hypothesized that genomic regions corresponding to chromatin states not well represented in the training data could be associated with reduced correlations. To test this, we analyzed the genomewide score correlations of selected pairs of expert predictions after excluding genomic positions corresponding to individual or pairs of chromatin states (Supplementary Figure S4). We focused on two expert pairs with the lowest pairwise correlations (Gasperini/CRISPR/K562 and Wang/STARR-seq/GM12878; Gasperini/CRISPR/K562 and Kheradpour/MPRA/K562) and two expert pairs that were of the same cell or assay type (Ernst/MPRA/K562 and Gasperini/CRISPR/K562;

Gasperini/CRISPR/K562 and Fulco/CRISPR/K562) that had low correlations. We observed that removing particular combinations of transcription-associated chromatin states resulted in positive and higher correlations (Supplementary Figure S4). Notably, chromatin states 8_TxWk and 5_Tx5’ had zero loci tested within the Gasperini/CRISPR/K562 dataset (Supplementary Figure S2) and removing loci assigned to these states resulted in the largest correlation increases for the pairs that included Gasperini/CRISPR/K562.

**Supplementary Figure S4:** Genomewide score correlations for selected pairs of expert predictions after removing loci assigned to individual chromatin states or combinations of chromatin states. The selected pairs all had low or negative correlations before removing the loci, for which results are shown in bar-graphs, are **(A)** Gasperini/CRISPR/K562 and Wang/STARR-seq/GM12878, **(B)** Gasperini/CRISPR/K562 and Kheradpour/MPRA/K562, **(C)** Ernst/MPRA/K562 and Gasperini/CRISPR/K562, **(D)** Gasperini/CRISPR/K562 and Fulco/CRISPR/K562. Score correlations are averaged over 30 randomly selected cell types. The bar with the label “()” and the dashed red line indicate correlations with no chromatin states removed. Chromatin states in pairs of states for which removal of loci that led to the largest increase in correlation include the transcription associated chromatin states 5_Tx5, 6_Tx, 8_TxWk, 11_TxEnh3, and Heterochromatin associated state 21_Het.

**Reviewer 2**

The authors present a new approach for the prediction of active regulatory regions by integrating several types of genome-activity assays in a supervised framework, in which histone mark data is used as features for regression. The ChromScore and subsequent ChromScoreHMM is designed as a way to integrate and interpret histone marks in regulatory regions. The authors present interesting results regarding their new approach and compare to other existing methods that score regions of relevance as well as a thorough analysis of their new predictions. Albeit complex, the paper is generally well written.

**Response:** We thank the reviewer for the summary and the positive and constructive comment. We provide a point by point response to the individual comments below.

**Major comments:**

- The authors use imputed signal tracks for 10 histone marks and Dnase1 for the learning of the ChromActivity epxerts. In how far do the results differ if real tracks are being used? Is the prediction performance similar for imputed and real histone tracks?

**Response:** We thank the reviewer for the question. In response, we have evaluated cell type generalization predictive performance for a model trained on observed signal tracks part of the Roadmap Epigenome Consortium processing (Supplementary Figure S19) and found the performance to be indeed similar to a model trained on imputed data. As the set of chromatin state annotations between the observed and imputed data differ, we restricted this analysis to use only signal data to make a more direct comparison which we would expect to have minimal effect on the overall results (see response to next comment). Overall we observed essentially the same predictive performance on average between observed and imputed data.

We have updated the “ChromScore regulatory activity predictions” section in Results and “Evaluating ChromScore cell type generalization performance” section in Methods to include the new analysis.

We also compared ChromScore predictions when training and using features based on observed data rather than imputed data, which yielded equivalent cell type generalization performance (Supplementary Figure S19).

**Supplementary Figure S19: Cell type generalization performance of the ChromScore model trained on imputed vs. observed Roadmap Epigenomics dataset for each evaluation dataset.** Each pair of bars shows the AUROC for imputed and observed data for one functional testing dataset with the last pair of bars showing the average AUROC over all the datasets. Both models achieved equivalent average AUROCs across the evaluation datasets: 0.758 for the model trained on imputed data and 0.758 for the model trained on observed data, with small differences across individual evaluation datasets. To enable direct comparison between models, chromatin state features were removed during training of both models.

**Methods: Evaluating ChromScore cell type generalization performance**

To evaluate cell type generalization performance of the ChromScore model trained on observed rather than imputed data, we used observed histone modification signals and peak annotations processed by the Roadmap Epigenomics consortium [9] for data for the cell types K562, HepG2, A549, GM12878, HeLa-S3 originally generated by the ENCODE Consortium. All of these cell types had observed data for all marks in addition to a functional testing dataset we considered. We excluded chromatin state features during training so the models would be more directly comparable between observed and imputed data. Consistent with our previous approach, we removed experts trained on cell types matching the evaluation cell type.

-As the authors use so many tracks as input I wasn't able to understand, how the method could be used by others? Does a user always have to give the histone, DNase and CHrommHMM state tracks as input? Are all of those features equally important? It is quite a lot of input features that a user would have to prepare.

**Response:** We thank the reviewer for the questions. We first note that in many cases we expect a user to directly use existing ChromActivity annotations for downstream analyses. However, for cases where a user would like to generate new ChromActivity annotations, using the same set of features is not required. ChromActivity can be trained on any combination of histone, chromatin accessibility or other annotations that are available, however the performance of the models could vary.

To better understand the predictive performance of smaller subsets of features, we have added an analysis to the manuscript evaluating predictive performance when using subsets of features that we considered here (Supplementary Figure S20). We found that removing individual features linked to signal and peak of specific marks or removing all peak call or chromatin state features had a small effect on AUROCs (0.73-0.74, compared to 0.76). However, removing all signal features led to a more substantial decrease in prediction performance (0.70). These results suggest that it would be possible to use a smaller set of features than we used here and obtain comparable predictive performance, but also the value of at least having multiple signal features.

We also note and related to the previous comment above it is possible to generate predictions using imputed data, which is an option when marks are available in only subsets of samples.

**Supplementary Figure S20:** Feature ablation study on cell type generalization performance. **(A)** Cell type generalization AUROCs averaged across all evaluation datasets after removing signal tracks and peaks for the mark as well as chromatin state annotations, which include the mark as a feature. **(B)** Cell type generalization AUROCs averaged across all evaluation datasets after removing all peak, chromatin state or signal features.

We updated the “ChromScore regulatory activity predictions” subsection to include the new analysis:

We investigated the relative importance of subsets of features on cell type generalization performance with a feature ablation study (Supplementary Figure S20). Removing individual features linked to specific marks, removing chromatin state features or removing peak features all had small effects on AUROCs (0.73-0.74, compared to 0.76). However, removing all signal features led to a more substantial decrease in prediction performance (0.70). These analyses suggest that it would be possible to use a smaller set of features than we used here and obtain comparable predictive performance, but also the value of at least having multiple signal features. Removing DNase signal tracks, DNase peaks calls, and chromatin state annotations, which were in part-based on DNase signal tracks, from the model leads to only a modest decrease in cell type generalization performance, indicating that the model's overall performance has limited dependence on DNase-based features.

- In the context of the evaluation and comparison of ChromScore performance (Fig 4B) I would be interested to understand better how the difference between CRISPR and Plasmid based assays can be understood. In Supp Fig S15 A, it shows that ChromScore works relatively poor for CRISPR-based assays in comparison to many other established methods and even the single histone tracks. How can this be? For Plasmid based assays (SUpp Fig S15 B, it looks quite different).

**Response:** We thank the reviewer for the question. Due to the distinct characteristics of plasmid-based and CRISPR-based assays, we would expect experts trained on CRISPR-based assays to perform better when predicting CRISPR-based assay outcomes. We believe since our specific application of ChromActivity included more plasmid-based datasets than CRISPR-based datasets (leading to a higher weight on plasmid-based assays in the ChromScore calculation), we observed lower performance on CRISPR-based datasets. Our

evaluation of individual experts' predictive performance (Supplementary Figure S15), which shows that the CRISPR-based expert achieves the highest performance when predicting the other CRISPR-based assay, supports this view. Future applications of ChromActivity that include additional CRISPR-based datasets or assign larger weights CRISPR-based experts could improve performance in CRISPR-based assays.

We added a sentence to the Discussion section about this point:

Future extensions of ChromActivity could assign larger weights to CRISPR-based experts or include additional CRISPR-based datasets could improve performance in CRISPR-based assays.

-Similarly Fig 4B shows that the individual DNase1 track alone works almost as good as the complete ChromScore approach. The authors write that the correlation with DNAse1 signal is only moderate, which may argue for some type of complementarity. It would be great if the authors could explore this in more detail. For example using true positive cases, is there something specific about the regions that are found with DNAse1 or vice versa?

**Response:** We thank the reviewer for their question. In Supplementary Figure S18, we compared chromatin state fold enrichments of top ChromScore and DNase-I hypersensitive regions and observed considerable differences, particularly for the 20_ZNF/Rpts (18.01 vs. 0.11 fold), 2_PromU (12.19 vs 29.20 fold) and 3_PromD1 (7.08 vs. 34.05 fold) states. We have updated the “ChromScore regulatory activity predictions” in the Results section to indicate this finding:

Furthermore, the chromatin state distributions of top ChromScore regions differed considerably from top DNase regions (Supplementary Figure S18) particularly for the 20_ZNF/Rpts (18.01 vs. 0.11 fold), 2_PromU (12.19 vs 29.20 fold) and 3_PromD1 (7.08 vs. 34.05 fold) states.

- In the methods section (page 31, lines 523-525) the authors mention that they excluded repressive sequences from the training. I would like to understand the rational for this. Did it show improvement for the learning?

**Response:** We thank the reviewer for raising this point. Repressive data points were only available on a subset of the datasets. We chose to focus on activation so that we could include additional datasets that did not measure repression. To clarify this rationale we have added the following remark in the “Dataset selection and label extraction” subsection in Methods:

A subset of the datasets reported repressive sequences in addition to neutral and activating (Ernst/HepG2, Ernst/K562, Kheradpour/HepG2, Kheradpour/K562). For these datasets, we excluded these reported repressive regions while training the corresponding expert for consistency with other datasets that did not measure repression.

**Minor comments:**

- I find the use of the word ChromActivity as an overall name a bit confusing in the wordings sometimes. With ChromActivity the authors sometimes describe the supervised learning part of individual assays, and sometimes more generally the framework that can do many things. Maybe the authors could check the text carefully to avoid ambigous mentions (such as page 10, line 139: In addition, ChromActivity averages ...).

**Response:** We thank the reviewer for the feedback. We intended ChromActivity to refer to the general framework. We revised the manuscript to refine the language in the example provided by the reviewer and two additional instances we identified where the ChromActivity term was ambiguous.

The revised text now states:

As a first step in the ChromActivity framework, a separate model is trained for each functional characterization dataset to predict the relative likelihood of each 25-bp genomic interval showing activity based on chromatin mark features across the entire human genome.

In addition to the ChromScoreHMM annotations, the ChromActivity framework includes a final step where predictions from different experts are averaged to generate ChromScore, a single genomewide regulatory activity potential score for each cell type.

In the supervised learning component of ChromActivity, ChromActivity uses a bagging ensemble of regularized logistic regression classifiers to generate the individual experts, which has the advantages of being robust and providing well-calibrated probability estimates that reflect the class membership of the training data.

- Figure 2 B, denotes the names of ChromHMM states on the x-axis, but these names are not explained anywhere. Please add a description to the paper (possibly in the legend of Fig2) so that the reader can follow. Supp Fig. S2 explains the color code but not the meaning of the abbreviations. And personally I don't find it helpful to have the color code legend in the supplement.

**Response:** We thank the reviewer for the feedback. We have added explanations for the ChromHMM state names to the Figure 2 legend. We also updated the legend to indicate that the color of the boxes within the boxplot correspond to each ChromHMM color, as we believe this communicates both score distributions and ChromHMM colors efficiently. For ChromScoreHMM, we also have the colors on the left side of the emission parameter matrix in Figure 3A.

Figure 2 legend updates:

**(B)** Box colors correspond to the predefined ChromHMM imputed 25-state model colors.

Chromatin state abbreviations: active promoters (1_TssA, 2_PromU, 3_PromD1, 4_PromD2), transcribed regions (5_Tx5, 6_Tx, 7_Tx3, 8_TxWk), transcribed & regulatory regions (9_TxReg, 10_TxEnh5, 11_TxEnh3, 12_TxEnhW), active enhancers (13_EnhA1, 14_EnhA2, 15_EnhAF), weak enhancers (16_EnhW1, 17_EnhW2, 18_EnhAc), primary DNase (19_DNase), ZNF genes & repeats (20_ZNF/Rpts), heterochromatin (21_Het), poised/bivalent promoters (22_PromP, 23_PromBiv), repressed polycomb (24_ReprPC), and quiescent/low (25_Quies).

- I was not able to understand how the Genome % (Annotations) row is defined in Figure 3B (top row above the heatmap). Please extend the Figure caption for it as the current description "percentage of the genome occupied by the annotation" is ambiguous. Currently the numbers do not add up to 100%.

**Response:** We thank the reviewer for pointing out this was unclear and we apologize for that. We updated the legend to clarify this row. The different annotation categories can overlap which is the reason why they add up to a value above 100%.

Figure 3 legend change:

**Top row** ("Genome % (Annotations)"): Percentage of the genome covered by each annotation type, calculated by dividing the number of genomic base pairs covered by a given annotation by the total size of the genome. As some annotations can overlap each other, the sum of these percentages exceeds 100%.

- I prefer the visualisatoin in Supp Fig S1 for teh ChromActivity framework over what is currently in Fig 1A, as it contains more details and also helps to understand the difference between ChromScore and ChromscoreHMM. I suggest that the authors integrate it into the main paper.

**Response:** We thank the reviewer for the feedback. Based on this feedback we have made what was Supplementary Figure S1 our main Figure 1A.

Figure 1 and legend change:

**Figure 1: Overview of the ChromActivity framework. (A)** Flowchart of the ChromActivity framework. ChromActivity takes as input regulatory activity labels from targeted genomic regions from *k* different functional characterization datasets (stacked white blocks, upper left). Using features based on chromatin mark signals, peak calls and chromatin state annotations for the targeted regions (red block, lower left) which it preprocesses (purple block, lower left), it trains a separate classifier (“expert”) for each functional characterization dataset. Each expert provides a predicted genomewide regulatory activity score track specific to a functional characterization dataset (stacked blue blocks). ChromActivity then uses the score tracks to generate two complementary outputs reflecting predictions of regulatory activity for each cell type (yellow blocks, right): **(i)** ChromScoreHMM annotations, which are annotations of the genome into states generated by integrating combinatorial and spatial patterns in the expert prediction score tracks using ChromHMM and **(ii)** ChromScore tracks, which are continuous genomewide regulatory activity score tracks based on the mean individual expert scores at each 25-bp interval. **(B)** Visualization of regulatory activity score tracks for each expert, ChromHMM chromatin state annotations (25-state imputed model), the ChromScore track and ChromScoreHMM annotations in HepG2 for genomic interval chr1:6,000,000-6,100,000 (hg19). ChromScoreHMM and ChromHMM color legends are shown in Supplementary Figure S1.

**Response letter works cited**

Ernst,J. *et al.* (2011) Mapping and analysis of chromatin state dynamics in nine human cell types. *Nature*, **473**, 43–49.

Fulco,C.P. *et al.* (2019) Activity-by-contact model of enhancer–promoter regulation from thousands of CRISPR perturbations. *Nat. Genet.*, **51**, 1664–1669.

Liu,Y. *et al.* (2017) Evidence of reduced recombination rate in human regulatory domains. *Genome Biol.*, **18**, 193.

**2^nd^ round**

**Reviewer 1**

The revision has statisfatorily addressed all my concerns.

**Reviewer 2**

The authors have significantly improved the paper in the revised version and have addressed all comments appropriately.
